# A Paper Read before the Odontological Society, of Atlanta, Ga., at the December Meeting

**Published:** 1895-04

**Authors:** D. S. Arnold

**Affiliations:** Atlanta, Ga.


### Southern Medical Record.

A MONTHLY JOURNAL OF MEDICINE AND SURGERY.

VOL. XXV. ATLANTA, GA., APRIL, 1895. No. 4.

### Original Articles.

### A PAPER READ BEFORE THE ODONTOLOGICAL SOCIETY, OF ATLANTA, GA., AT THE DECEMBER MEETING.

BY DR. D. S. ARNOLD, ATLANTA, GA.

In the duty devolving upon me to provide a paper for this meeting of our society, I hope I may not be considered presumptuous in attempting to discuss a subject so important as the treatment of Fractured Jaws. The motive which prompted me to select this as my subject, was not a wide experience in treating fractured jaws, nor anything very new or original I had to offer concerning the management of such cases. But since beginning the practice of dentistry, it is a subject in which I have always taken a deep interest and one that is of great worth to every practitioner of our beloved profession.

The force which produces the fracture of the upper jaw must, as a rule, be direct and of great violence. A kick from a horse, a blow from some machine, the impact of a pistol ball, are among the most common causes of this injury. The fracture is often comminuted and is sometimes followed by concussion of the brain, or with fracture of other bones of the face, or at the base of the skull.

Associated as the upper jaw is, with no less than nineteen bones, such results create no surprise. This extensive connection of the superior maxillary will explain the modus of the singular accident presented by Packard where a man's head was caught between a hoisting machine and the floor, causing the entire separation of the face from the cranium. Fractures of the upper jaw are often caused by force transmitted from the malar bone, the malar bone remaining sound. This portion of the face being prominent, is likely to receive the vulnerating body first. But the bones most likely to participate in fracture of the superior maxilla are the malar, the nasal, the palatine and the pterygoid processes of the sphenoid. A cardinal principle to be observed in the treatment of all the fractures of the superior maxilla is to preserve and replace all fragments and splinters; the tendency to heal in this bone being greater than in any other bone of the skeleton. That the best results may be obtained, it is necessary for the dentist to have a perfect knowledge of the normal relations of the jaws and teeth and a most thorough familiarity with all appliances calculated to produce the best results, and at the same time give the most comfort to the patient. The general surgeon is invariably called upon to attend these cases. But now the more enlightened ones request that a dentist be also called, for he at once recognizes his inability to overcome the mechanical difficulties that may be presented in the case at hand. It is to our profession that all honor is due for the wonderful progress made in the treatment of fractured jaws, for the most approved methods and best appliances have emanated from dentists, and as a specialty in surgery belongs entirely to the dental surgeon. The fractures of the lower jaw are divided into the body, ramus, and processes, and are generally caused by direct force either upon the side of the bone or upon the chin. When involving the body they may be situated at any point from the angle to the symphisis of the bone, they may extend through the entire thickness of the bone or be confined to the alveola process; generally there is not much difficulty in determining a fracture of the lower jaw, although when the separation is incomplete it may not be so easy. There is a fixed and deep pain which is generally increased by any movement which disturbs the quietude of the jaw, as in opening and closing the mouth, swallowing or even taking a deep inspiration. We might suppose that the Inf. dental nerve from its position would frequently suffer damage in fractures of this bone, and consequently give rise to erious trouble; and yet such injury is almost unheard of. Swelling of one and more of the salivary glands is occasionally met with and may end with an abscess opening into the mouth or upon the neck. But fractures of the Inf. maxilla usually do well when the proper treatment is given. Tardy union is not uncommon, but non-union is very rare. It is believed that saliva has some influence in retarding the union of the bone. And the presence of the smallest particle of necrosed bone will defeat the healing process. The diagnosis of a fractured jaw, is unmistakable, the most prominent symptoms being pain, swelling, and crepitation, and hemorrhage where displacement of the teeth has occurred. When any doubt exists as to location of the fracture in the lower jaw, grasp the bone on each side, and then introduce the index fingers into the mouth, the fingers resting upon the teeth, and with slight in-and-out movement, you will locate the crepitation at the point of fracture. In the upper jaw use the thumbs instead of the index fingers. Fractures are simple when only the bone is divided without piercing the integuments. Compound when laceration is present and direct communication between the outside air and the fracture. Comminuted when the bone is broken or crushed into several pieces at the same point and communicating with each other. Fractures of the Inferior Maxilla if the body of the bone is involved are almost always compound in the direction of the mouth; but when the Ramus, coronoid process or condyle, are fractured the bone is too deeply seated for the injury to extend into the mouth, unless caused by a gun-shot or missile. Teeth that are loosened or fractured should not be taken out for they nearly always become firm and useful afterwards. But should a nerve be exposed in a tooth by an accident, it is best to destroy it, so as not to cause the patient any unnecessary pain. Before taking the impression for an inter-dental splint, always carefully remove any tartar that may be around the teeth, or else you will have as a consequence a loose fitting splint. You now take impression of both jaws, always taking the impression of the uninjured jaw first, so as to reassure the patient that it will not be painful.

And for impression material, I always use modeling composition, as plaster Paris is objectionable to the patient, and will invariably get into the lacerations, if any be present. I favor

Dr. Gunning's method of taking an impression; that is, to hold the jaw as near the normal position as possible, sawing your model after, at the fractured point, and articulate it with the upper teeth, re-cementing it with plaster. But should any mistake occur in the articulation, the failure of your splint is assured. So, you necessarily have to be very careful in the articulation of your divided model with the upper jaw. Fractures of the Superior Maxilla require but little treatment, compared with those of the lower, because the bones are immovable, and you have no trouble in keeping the fractured parts together. I always make my splints for the lower jaw, so as to prevent the use of the jaw, in any way whatsoever, by the patient, for in all cases that I have seen, where the jaw was used to masticate, I have seen invariably follow an excessive amount of bone, to deform the patient for life—an osseous tumor at the point of fracture, that only an operation will remove, and this is caused by the continued irritation that follows the constant slight rubbing together of the points of the fractured bone in masticating.

I will now briefly report two cases that I have recently treated with success. The first case I will present is that of a bar-keeper, forty-five years old, who had been shot with a 44 Colt's pistol, at close range, the ball entering the left side of the lower jaw, between the third molar and the first bicuspid, plowing out the intervening teeth and bone. The ball I found buried under the tongue, having greatly lacerated the soft tissues of the mouth. After removing all the small particles of bone, and the bullet from the lacerated parts, I held the jaw as nearly as I could in the normal position, and took the impression with modeling compound. I, of course, took the upper jaw first, and, after getting my model ready, I took my saw and cut the model of the lower jaw midway the point of fracture, and carefully articulated the fractured model with the upper teeth (which I had no trouble in doing). I then reunited my broken model with plaster. My splint covered all the lower teeth on both sides, and made so as to touch the upper teeth on both sides and in front; putting this in the mouth, I carefully secured the jaw with a four-tail bandage. In three weeks, I removed the splint, and found a beautiful reformation of the lost bone, having no excessive bone whatsoever, and to-day, but for the small scar on the outside, you could not tell he had ever been shot. I replaced the lost teeth with bridge-work.

The other was a machinist, twenty-four years old. He was standing by a machine, and a piece of heavy timber was caught in one of the wheels, and, swinging around, suddenly struck him with the sharp edge on the chin, knocking the six anterior teeth of the Inferior Maxilla imbedded in the alveolar process, back in his mouth, making a complete separation.

I simply took the pieces, and, by a little work, succeeded in getting it back in place, and beginning with the bicuspid, at the right side, I wired all the intervening teeth around to the corresponding tooth on the left side, and an uninterrupted union followed, the patient using his jaw all the time for mastication, and, strange to say, all the displaced teeth presenting every indication of possessing nervous sensation. I am firm in the belief that the nerves reunited, and are now in a healthy condition.

---

OPERATION FOR DEPRESSED NOSE.—Ellison (*Lancet*, February 17, 1894) gives the history of a case of depressed nose, in which a metal plate was inserted. He describes the procedure as follows: An incision with a tenotomy knife was made on either side and across the nose a couple of lines below the lower part of the depression. The flap of integument thus marked out was raised sufficiently to permit the plate to rest in the position desired on the subjacent tissues. There was very little bleeding, which was easily stopped with hot water. The flap of integument was then drawn over the plate and carefully sutured with horse-hair along the line of the incision; a thread of catgut as a drain was inserted in the lower portion of the incision, and removed after twenty-four hours. Collodion, except over the catgut drain, and gauze was applied as a dressing. Healing occurred by primary union. The patient seven years later stated that she had no incovenience from the plate whatever, thus establishing the complete success of the operation.

